# Central Nervous System Metastases from Primary Lung Carcinoma: Significance of RNA Fusion Testing and Early Versus Late Metastases

**DOI:** 10.3390/jpm15050181

**Published:** 2025-05-01

**Authors:** Michelle Garlin Politis, Mahesh Mansukhani, Benjamin O. Herzberg, Lanyi N. Chen, Mark Stoopler, Maelle Saliba, Markus Siegelin, Zhe Zhu, Joshua Sonett, Brian S. Henick, Simon K. Cheng, Swarnali Acharyya, Catherine A. Shu, Michael L. Miller, Benjamin Izar, Helen Fernandes, Susan Hsiao, Anjali Saqi

**Affiliations:** 1 Department of Pathology and Cell Biology, Columbia University Irving Medical Center, New York Presbyterian Hospital, New York, NY 10032, USA; mg4384@cumc.columbia.edu (M.G.P.); mm322@cumc.columbia.edu (M.M.); ms4169@cumc.columbia.edu (M.S.); hf2340@cumc.columbia.edu (H.F.); sjh2155@cumc.columbia.edu (S.H.); 2 Division of Hematology and Oncology, Columbia University Irving Medical Center, New York Presbyterian Hospital, New York, NY 10032, USA; 3 Department of Pathology and Laboratory Medicine, Memorial Sloan Kettering Cancer Center, New York, NY 10065, USA; salibam@mskcc.org; 4 Division of Thoracic Surgery, Columbia University Irving Medical Center, New York Presbyterian Hospital, New York, NY 10032, USA; js2106@cumc.columbia.edu; 5 Division of Radiation Oncology, Columbia University Irving Medical Center, New York Presbyterian Hospital, New York, NY 10032, USA; sc3225@cumc.columbia.edu

**Keywords:** CNS metastases, RNA fusion testing, NGS, lung carcinoma

## Abstract

**Background/Objectives:** While the genomic landscape of primary lung carcinomas is well characterized, there is a relative scarcity of fusion data on corresponding central nervous system (CNS) metastases. This study aimed to elucidate the molecular profiles of CNS metastases to (1) assess the significance of a combined DNA–reflex RNA fusion testing approach and (2) compare the mutational landscape between patients who present initially [early (≤2 months)] with CNS metastases and those who develop CNS metastases thereafter [late (>2 months)]. **Methods**: We performed a retrospective search of CNS metastases of adenocarcinoma of probable lung origin interrogated by targeted DNA–reflex RNA next-generation sequencing (NGS). The DNA NGS panel included the driver mutations *EGFR*, *BRAF*, *KRAS*, *MET*, and *ERBB2*. RNA NGS included *ALK*, *RET*, *ROS1*, and *MET*. Additionally, mutational profiles were examined between those with early versus late CNS metastases. **Results:** Of the 58 patients, 44 (75.9%) had mutations or alterations, including 34 identified by DNA NGS [*EGFR* (*n* = 17; 50.0%), *KRAS* (*n* = 15; 44.1%), *MET* (*n* = 2; 5.9%)] and 10/24 by RNA NGS [*ALK* (*n* = 7; 70%), *MET* (*n* = 2; 20%), *ROS1* (*n* = 1; 10%)]. Of all patients, 32 (55%) presented with early and 26 (45%) with late CNS metastases. Although patients with early metastases had worse survival compared to those with late metastases (*p* < 0.001), there were no statistically significant differences in the mutational profiles between the two cohorts. **Conclusions**: A significant proportion of CNS metastases without driver mutations identified by DNA NGS had targetable alterations identified by RNA NGS (10/24, 41.7%). In summary, a combined DNA with reflex RNA fusion testing approach plays a significant role in detecting and potentially managing CNS metastases. Comprehensive prospective studies are essential to elucidate the differences between early and late CNS metastases.

## 1. Introduction

Lung cancer is the leading cause of cancer-related deaths worldwide [1]. Metastatic non-small cell lung cancer (NSCLC) to the central nervous system (CNS) has been historically associated with a guarded prognosis. However, mortality from NSCLC has been declining, in part due to the advent of targeted therapies for driver mutations such as *EGFR* and *MET* (*exon 14*) and fusions such as *ALK*, *ROS1*, *NTRK1/2/3*, and *RET* [2]. A recent report demonstrated a promising overall survival (OS) of 2.5 years among patients with CNS metastases from lung adenocarcinoma [3]. Targeted therapies have been shown to improve CNS disease control and patient survival for NSCLC with actionable genomic alterations like *EGFR* and *ALK* [4,5,6]. For instance, the tyrosine kinase inhibitor (TKI) lorlatinib, which targets *ALK* rearrangements, demonstrated a median progression-free survival (PFS) exceeding 5 years, and the vast majority of patients with CNS metastases experienced complete and durable response. Meanwhile, multiple other mutational targets are under investigation in clinical trials [7].

The improved OS and PFS data with targeted treatments for driver mutations underscore the significance of comprehensive genomic testing in NSCLC. While single nucleotide changes and indel driver mutations identified by DNA next-generation sequencing (NGS) are present in 60 to 80% of lung adenocarcinomas, fusions identified by RNA NGS represent a smaller but significant fraction [8].

Regardless of the known significance of genomic profiling, up to 64% of patients with NSCLC may not benefit from precision oncology therapies due to various clinical practice gaps, such as not having the appropriate biomarker testing ordered or initiating treatment before the availability of test results [9,10]. Furthermore, some NSCLC cases undergo only DNA NGS, whereas RNA NGS could potentially identify actionable alterations in carcinomas that lack driver mutations detected by targeted DNA NGS [11,12].

Notably, lung carcinomas exhibit the highest rate of CNS metastases relative to carcinomas from other sites, and over 50% of patients present initially with CNS metastases [13]. In general, metastases are classified as synchronous or metachronous based on the time of presentation. Previous studies of CNS metastases have separated synchronous and metachronous at 2 months based on potential biological aggressiveness and availability of epidemiological data [13,14,15]. CNS metastases have also been evaluated in the context of a third precocious category, in which the metastases represent the initial presentation of the primary carcinoma [15,16]. We categorized precocious and synchronous as early (presentation prior to or within 2 months of identifying the primary carcinoma) and metachronous as late (presentation > 2 months following primary carcinoma diagnosis). There are limited data concomitantly comparing early and late presentations of lung carcinoma CNS metastases in the context of DNA and RNA NGS genomic profiling.

This study aims to evaluate the mutational signatures of CNS metastases and (1) elucidate the role of targeted DNA NGS with reflex RNA NGS for the identification of gene fusions and (2) characterize the mutational landscapes of early (CNS metastases as initial presentation or <2 months from the lung primary carcinoma) and late (CNS metastases > 2 months following the initial diagnosis of lung carcinoma) CNS metastases.

## 2. Materials and Methods

**Figure 1:**
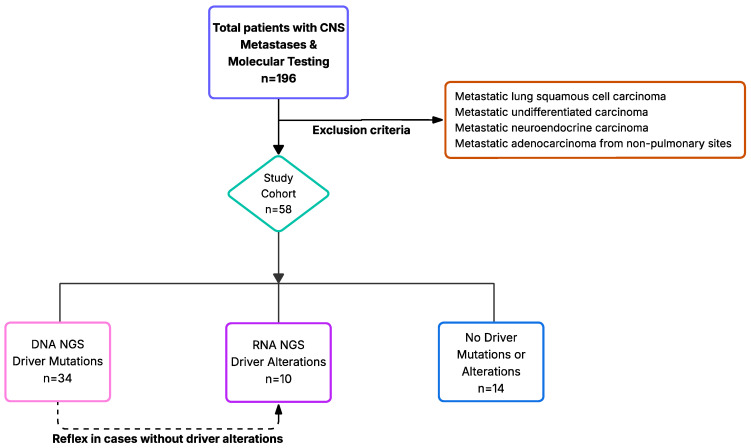
Study Cohort Selection: CNS metastases with adenocarcinomas of (probable) lung origin with sufficient tissue for DNA and RNA NGS.

Following Institutional Review Board (IRB) approval, pathology records were retrospectively searched (7 November2018–21 April 2023) to identify all specimens with CNS metastasis and adenocarcinoma of (probable) lung origin or when this diagnosis could not be excluded. Inclusion criteria were sufficient tissue for DNA and RNA NGS (Figure 1). The molecular profiles of the CNS metastases were compared with lung carcinomas profiled in our institution during the same timeframe and fulfilled identical inclusion criteria.

### 2.1. Targeted NGS and Sequencing Data Processing

The DNA NGS panel included targeted regions in 13 genes. The primary focus of this study was on genes recognized by National Comprehensive Cancer Network (NCCN) NSCLC guidelines in 2023, including *EGFR, KRAS, BRAF, ERBB2,* and *MET.* The specific regions tested were *BRAF* (NM_004333 e11,15); *EGFR* (NM_005228 e3,7,12,15,18–21); *ERBB2* (NM_004448 e8,17,19–21); *KRAS* (NM_004985 e2–4); *MET* (NM_001127500 e2,14,16,18,19). Variants in additional genes (e.g., *PIK3CA*, *KEAP1*, *POLD1*, *STK11*) were reported when detected.

Tumor samples submitted for testing were reviewed by a pathologist to confirm sufficient sample for testing, and microdissection was performed to enrich for tumors if necessary. Genomic DNA was extracted from formalin-fixed paraffin-embedded (FFPE) samples and libraries were prepared for sequencing as described previously [17]. In brief, 2.5–20 ng of tumor DNA was used to generate libraries using Pillar Biosciences’ proprietary SLIMamp™ (stem-loop inhibition mediated amplification) technology, with adapters for Illumina sequencing, followed by sequencing on the Illumina MiSeq platform with reversible fluorescent terminators. Reads were aligned with BWA-MEM, and variants were identified using Vardict and ABRA2. Annotation was performed using Annovar and a custom in-house developed pipeline. The limit of detection is 3% variant allele fraction. The minimum average coverage of the assay was 500X.

### 2.2. RNA NGS and Sequencing Data Processing

Identification of fusions or oncogenic isoforms was reflexively performed by RNA NGS in cases that lacked DNA tyrosine kinase-RAS-MAP kinase pathway driver mutations. The panel included 17 genes, but this study focused on fusions or oncogenic isoforms in genes noted in the NSCLC NCCN guidelines in 2023: *ALK*, *MET*, *ROS1*, *RET*, and *NTRK1/2/3*.

RNA from FFPE samples was extracted using the Qiagen AllPrep DNA/RNA FFPE kit and Qiacube protocol for FFPE total RNA. In total, 20–50 ng RNA was used for library preparation using Anchored Multiplex PCR (AMP™) technology (ArcherDx, Boulder, CO, USA), and multiplexed sequencing was performed with molecular barcode adapters and indexing for downstream sequencing on the Illumina MiSeq platform. Both known and novel fusion partners in key fusion genes were interrogated. Data analysis was performed using the Archer Analysis bioinformatics software (version 6.0.4).

### 2.3. MET Exon Skipping Mutations

The DNA NGS panel includes the targeted region for the identification of *MET* exon 14 skipping mutations. However, some *MET* exon skipping mutations may not be detected by DNA NGS due to a lack of complete coverage of intron 13 splice acceptor site, polypyrimidine tract, and branch point and must be identified with RNA NGS [18]. Only the primary detection method—DNA NGS or RNA NGS—of *MET* exon skipping mutations is included.

### 2.4. Early vs. Late CNS Metastases

The cohort was subdivided by the time of CNS metastasis presentation: either early (CNS metastases as the initial presentation or <2 months from the lung primary carcinoma) or late (CNS metastases >2 months following the initial diagnosis of lung carcinoma) in alignment with prior categorizations [13,14,15].

### 2.5. Clinical Characteristics and Survival

Patients’ demographics and characteristics were compared between those with and without driver mutations and alterations as well as between those with early and late CNS metastases. For patients with early and late metastases, the main factors prompting medical attention for the primary lung carcinoma were assessed: symptomatic, incidental, screening, or other. Survival curves were generated using the Kaplan–Meier method in the context of (1) any versus no mutations or alterations, (2) DNA mutations versus RNA alterations, and (3) early versus late CNS metastases.

### 2.6. Statistical Analysis

Categorical variables are expressed as percentages, whereas continuous variables are expressed as means or medians. Continuous variables conforming to normal distribution were analyzed using Student’s *t*-test or 1-way analysis of variance and are presented as mean ± standard deviation. Continuous variables not conforming to normal distribution were analyzed using the Mann–Whitney U test or Wilcoxon rank sum and are presented as median ± interquartile range. Categorical data were tested using the chi-squared test or Fisher’s exact test. A *p*-value < 0.05 was considered statistically significant. All statistical analyses were performed with SPSS (IBM Corp. 2017. IBM SPSS Statistics for Mac, Version 25.0. Armonk, NY, USA: IBM Corp.).

## 3. Results

### 3.1. Patients with Driver Mutations

In total, 58 patients fulfilled the inclusion criteria and comprised the study cohort. The patients were categorized as having or not having a detectable driver mutation or alteration (Figure 1). Most patients were male (53.4%), and most were either former or current smokers (72.7%) (Table 1).

Upon stratifying patients based on the presence or absence of variants detected by DNA NGS or RNA NGS, individuals with any alterations exhibited a non-significant trend towards a lower smoking history compared to those without mutations [(27/44) 43.6% vs. (13/14) 92.9%, *p* = 0.05)] (Supplemental Table S1). Additionally, those without mutations had a trend of self-reported white race [(9/14), 75% vs. (17/44) 43.6% with mutations (*p* = 0.06)] (Supplemental Table S1). There were no significant differences in other evaluated demographics (Supplemental Table S1).

**Figure 2:**
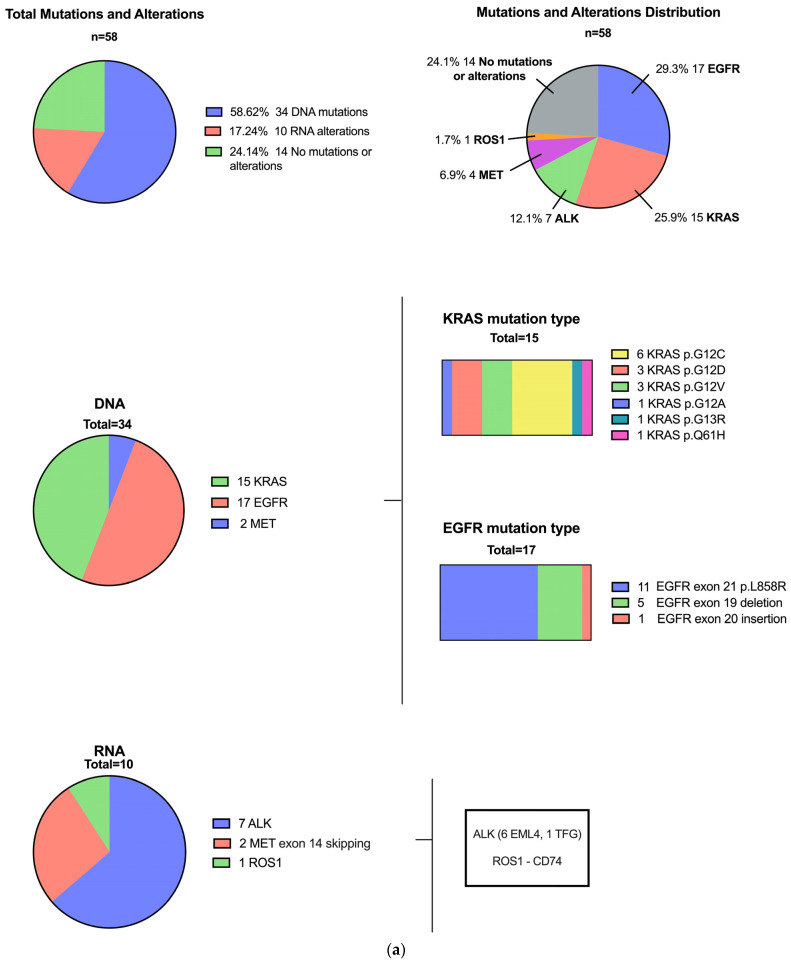

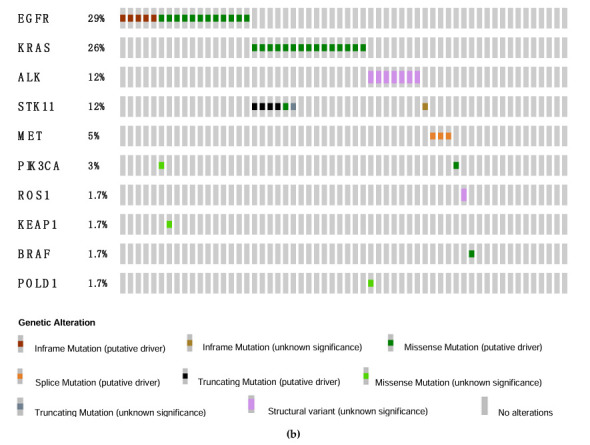
(**a**). CNS Mutation Profiles in All Patients: DNA NGS and RNA NGS. (**b**). Distribution of Key Gene Alterations in CNS Metastases in the Study Cohort in Order of Frequency. Each bar represents one patient sample, color-coded by mutation type. Percentages indicate the overall frequency of each gene alteration.

Of the 44 (75%) patients with driver mutations or alterations, 34 harbored DNA drivers (58.6% of the total sample): *EGFR* (*n* = 17), *KRAS* (*n* = 15), and *MET* (*n* = 2). Ten patients showed alterations by RNA NGS (17.2% of the total sample): *ALK* (*n* = 7), *MET* (*n* = 2), and *ROS1* (*n* = 1) (Figure 2a,b). During the same timeframe, 1550 patients with lung carcinoma were profiled in our institution, and 605 (39%) had driver mutations or alterations (Supplemental Figure S1).

Importantly, 10/24 (41.7%) patients without detectable mutations on the DNA panel exhibited targetable alterations on the RNA fusion panel. This resulted in benefits for one in 2.4 patients tested with the RNA fusion panel (Figure 2a).

**Figure 3:**
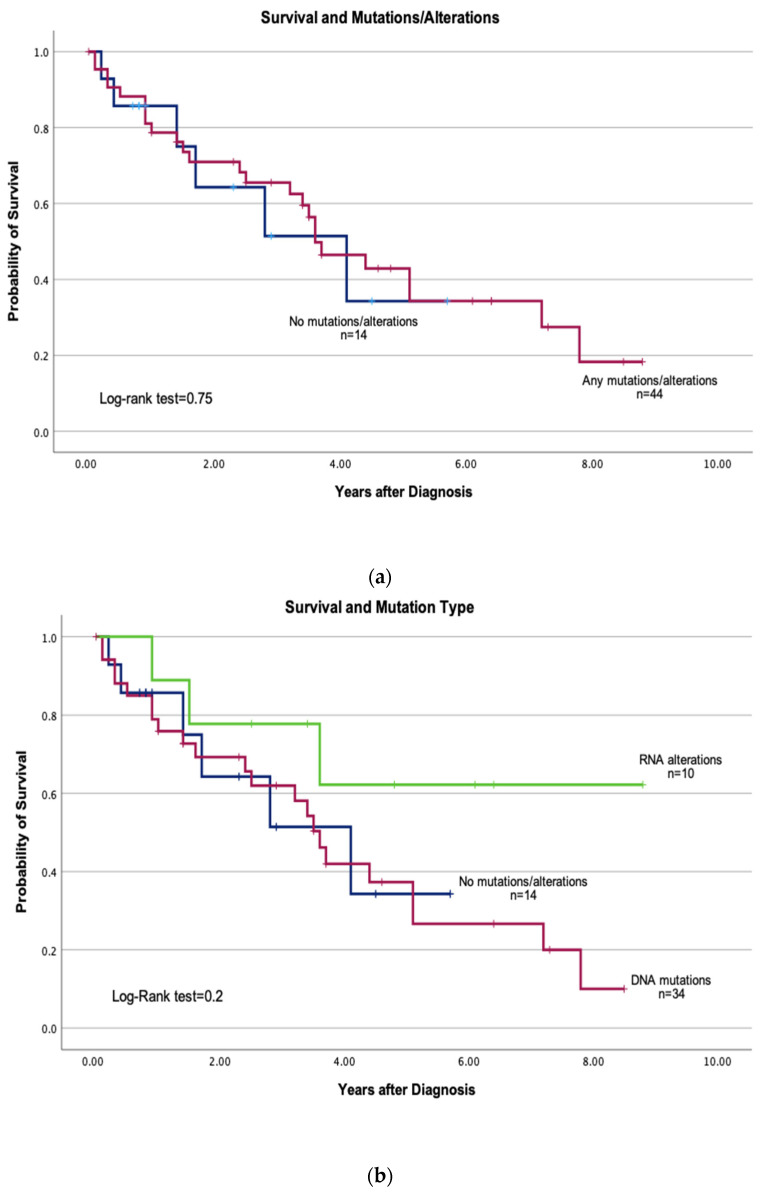
(**a**) Kaplan–Meier Survival Curves Comparing Patients with Mutations versus No Mutations or Alterations. The median survival for patients with mutations was 3.6 years (95% CI: 2.50–4.6), and for those without mutations or alterations it was 4.1 years (95% CI: 1.3–6.8). Log-rank test *p* = 0.75. (**b**). Kaplan–Meier Survival Curves Comparing Patients with DNA mutations, RNA alterations, and No Mutations or Alterations. The mean survival for patients with DNA mutations was 3.9 years (95% CI: 2.85–4.9), for those with RNA alterations it was 6.45 years (95% CI: 4.26–8.63), and for those without mutations or alterations it was 3.39 years (95% CI: 2.14–4.65). Log-rank test *p* = 0.2.

Survival analysis showed that patients with driver mutations or alterations (*n* = 44) had a median survival of 3.6 years (95% CI: 2.50–4.6), while those without mutations or alterations (*n* = 14) had a median survival of 4.1 years (95% CI: 1.3–6.8) (Figure 3a).

Upon stratification, patients with alterations detected by RNA NGS (*n* = 10) exhibited a non-significantly better survival rate of 6.45 years (95% CI: 4.26–8.63) (Figure 3b). In contrast, patients with mutations detected by DNA NGS (*n* = 34) had a median survival of 3.9 years (95% CI: 2.85–4.9), while those without mutations or alterations (*n* = 14) had a median survival of 3.39 years (95% CI: 2.14–4.65) (Figure 3b).

### 3.2. Patients with Early vs. Late CNS Metastasis

**Figure 4:**
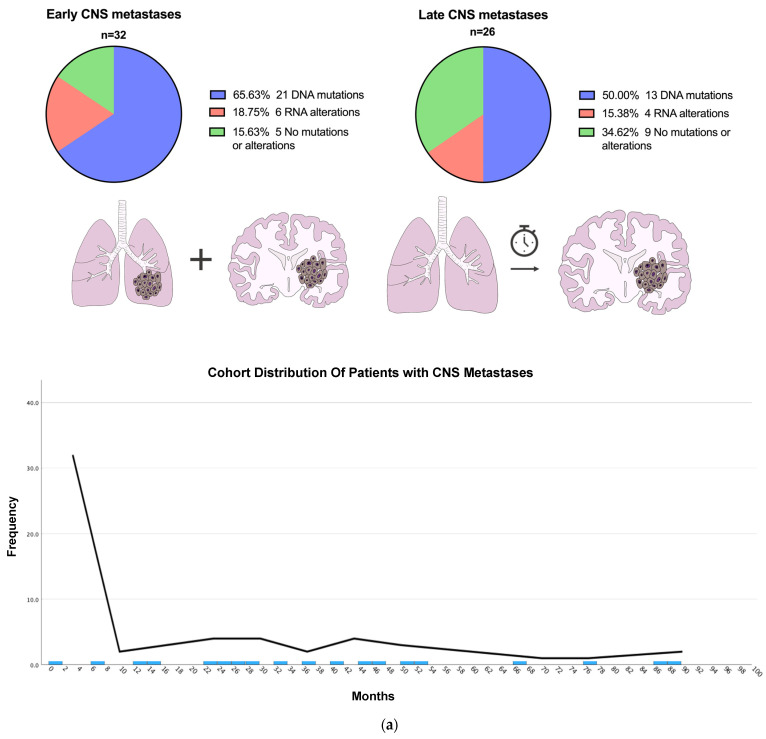

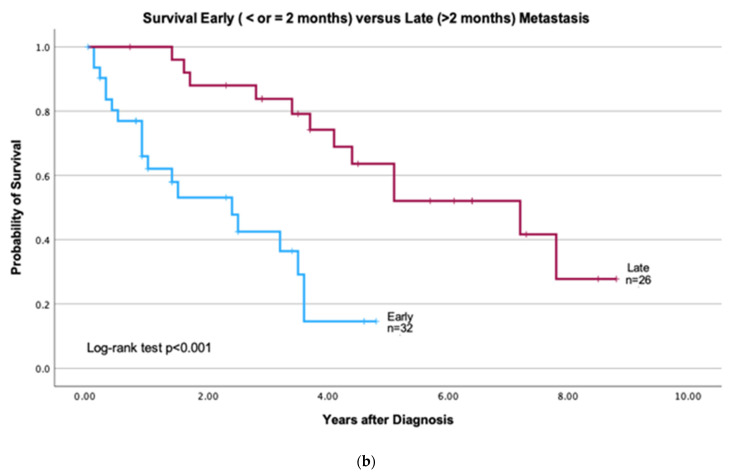
(**a**). Early versus Late CNS Metastases and Cohort Distribution. Profiles of DNA mutations and RNA alterations. The mean time for presentation of late metastases was 38.5 months (SD = 22.25), and the median was 33.2 months (IQR = 23.9). (**b**). Survival of patients with Early versus Late CNS Metastases. Kaplan–Meier survival curves for patients with early and late metastasis. The median survival was 2.4 years (95% CI: 0.99–3.81) for early metastasis and 7.2 years (95% CI: 3.96–10.45) for late metastasis. Censored observations (patients still alive at the last follow-up) are marked as ticks. Events and censored cases are mutually exclusive. Statistical significance was assessed using the log-rank test (*p* < 0.001).

The analysis demonstrated that 32/58 (55%) of the patients presented with early and 26/58 (45%) presented with late CNS metastases (Table 2, Figure 4a). The mean time for presentation of late metastases was 38.5 months (SD = 22.25), and the median was 33.2 months (IQR = 23.9).

Survival in patients with early metastases (*n* = 32) was significantly lower than in those with late metastases (*n* = 26) 10 years after diagnosis (*p* < 0.001). The median survival was 2.4 years (95% CI: 0.99–3.81) for early metastases and 7.2 years (95% CI: 3.96–10.45) for late metastases (Figure 4b).

There were no statistically significant differences in age, gender, or smoking status between patients with early and late CNS metastases. Self-reported white race was significantly associated with late CNS metastases (*p* = 0.021) (Supplemental Table S2. Patients’ Characteristics: Early versus Late mutations).

Clinical history prompting the initial presentation of the early CNS metastases or primary lung carcinoma in patients with late metastases was available for 30/32 and 18/26 patients, respectively. Most patients with early CNS metastases presented with CNS symptoms, and only two patients presented initially with respiratory symptoms. Of the 18 patients with late metastases, the primary lung carcinoma was incidental in 9 and symptomatic in 9. There were no patients with carcinoma detected by screening methods (Supplemental Table S2. Patients’ Characteristics: Early versus Late mutations).

Among those with early CNS metastases, 27/32 (84.4%) patients exhibited mutations or alterations, and 17/26 (65.4%) patients with late CNS metastases had mutations or alterations. The two most common mutations in early and late CNS metastases involved *EGFR* and *KRAS* genes: early *EGFR* (11/32; 34.4%) and *KRAS* (8/32; 25%); late *KRAS* (7/26; 26.9%) and *EGFR* (6/26; 23.1%) genes.

Notably, among early CNS metastases, six patients (60% of total RNA alterations) harbored gene fusions or *MET* exon 14 skipping mutations, specifically, four with *ALK* fusions and two with *MET* exon 14 skipping. Meanwhile, four patients with late CNS metastases exhibited gene fusions (three *ALK* and one *ROS1*).

### 3.3. Mutational Profile of Primary Lung Compared to CNS Metastatic Carcinomas and Additional Variants

Ten patients with primary lung carcinoma followed by CNS metastases had molecular data for both. These included two patients with early and eight patients with late metastases. Analysis of the primary lung carcinomas showed the following mutations: *EGFR* (six cases, five in late metastases: one exon 19 del and three L858R; one early metastasis: exon 19 del), *KRAS* (three cases, two in late metastases: G12D, G12V; one in early metastasis: G12C), and *ERBB2* (one case with late metastasis, S310F). The mutations were concordant between the primary and metastatic carcinomas in all, except for the *ERBB2* case, where the primary carcinoma had the mutation, while the corresponding metastasis showed no detectable mutations, possibly due to sampling bias or tumor heterogeneity.

Lastly, seven patients had *STK11* mutations that were frequently associated with *KRAS* mutations (Figure 2b). Six of these patients had both *STK11* and *KRAS* mutations: three had *STK11* P281fs, one had D194H, one had Q305*, and one had V116fs. One patient had an *STK11* K78_L80del mutation without other identifiable DNA or RNA mutations (Supplemental Table S3. Mutations Early versus Late CNS Metastases, Additional Variants).

## 4. Discussion

Our study demonstrates that primary lung carcinomas and CNS metastases have similar types of driver mutations and alterations [8]. Of 58 cases, 76% (44/58) harbored driver mutations that were detected by DNA NGS 34/44 (77%) and RNA NGS 10/44 (23%). RNA NGS proved beneficial for one in every 2.4 patients who did not have a driver mutation detected by DNA NGS. The frequency of DNA mutations and RNA alterations was non-statistically greater in early (84.4%) compared to late (65.4%) CNS metastases. These data emphasize the importance of performing both DNA and RNA NGS to identify driver mutations in CNS metastases and that reflex approaches may be a suitable and cost-effective strategy.

### 4.1. Mutational Profiles of Lung Carcinoma with CNS Metastases

Compared to the 39% of patients’ samples with driver mutations and alterations who underwent sequencing of lung carcinomas at our institution, 76% of patients with CNS metastases harbored driver mutations and alterations, predominantly *EGFR*, *KRAS*, and *ALK*.

Limited data specifically evaluating the mutational profiles of NSCLC CNS metastases corroborate some of our findings and indicate an increased prevalence of other alterations—*ROS1* in one cohort and *RET* in another. In a review examining the CNS metastases of lung carcinomas, Tan et al. reported a general incidence of CNS metastasis in patients with NSCLC of 10–20%. However, higher incidences were observed in patients with specific molecular alterations: 20–39% in patients with *ALK* fusions, 29% in patients with *KRAS* mutations, 23–32% in patients with *EGFR* mutations, and 19–36% in patients with *ROS1* fusions [19]. Moreover, in a meta-analysis of 24,784 patients with NSCLC, the prevalence of developing CNS metastases was 28.6% [20]. The prevalence of CNS metastasis in patients with specific genomic alterations was the following: *ALK* (34.9%), *RET* (32.2%), *KRAS* (30.2%), *EGFR* (29.4%), and wild type (28.8%) [20].

### 4.2. Significance of Combined DNA NGS and RNA NGS

In our sample, the combined approach of DNA NGS with reflex RNA NGS led to the reclassification of patients from having no targetable mutations to carrying clinically actionable mutations and alterations.

The significance of incorporating RNA NGS, which overcomes the shortcomings of DNA NGS to detect fusions, has been previously illustrated in a few studies. Benayed et al. identified alterations, including fusions and *MET* exon 14 skipping mutations, in 14% (36/254) of additional lung carcinomas that were negative by DNA NGS [11]. These findings enabled the matching of patients with targeted therapies, resulting in positive clinical outcomes. Similarly, Owen et al. identified 15.3% additional actionable structural variants with the addition of RNA NGS [12].

Multigene DNA testing is widely recognized and recommended in metastatic or advanced tumors. Meanwhile RNA NGS, which is also strongly recommended, may not be used as frequently [7,12]. Not all samples may undergo combined DNA and RNA NGS testing due to multiple factors, including inadequate specimen quantity, lack of familiarity with the increased likelihood of fusions (especially in CNS metastases), and lack of awareness of the differences in detection capabilities between DNA NGS and RNA NGS. Also, RNA NGS testing may be unavailable or cost prohibitive. Benayed et al. proposed a sequential workflow (RNA NGS for cases without driver mutations detected by DNA NGS), which may mitigate costs. However, others suggest concomitant DNA NGS and RNA NGS to avoid delays of several weeks in management [11,12].

It is worth noting that *ALK* alterations detected by other methodologies, including immunohistochemistry (IHC) and fluorescent in situ hybridization (FISH), are also eligible for targeted therapies. These methods offer a shorter turnaround time, greater accessibility, and a more cost-effective alternative to NGS but may occasionally have false positive results. In contrast to IHC and FISH, RNA NGS provides information on specific and novel fusion partners. While requiring further investigation, such information may provide greater insight into any association between specific fusion partners and the efficacy/resistance of targeted treatments [21].

### 4.3. Early vs. Late CNS Metastases

The main differences between the early and late cohorts were presentation (symptomatic vs. asymptomatic), survival, and race.

The distribution of early versus late CNS metastases in our cohort aligns with previously reported data [13]. Survival analysis revealed that patients with early CNS metastases had markedly worse outcomes. It remains unclear whether the differences between the two groups in our cohort are secondary to socioeconomic reasons, unrecognized genomic differences that highlight early CNS metastasis as an aggressive phenotype, or other factors.

The mutational profiles of CNS metastases of lung carcinoma have been described, but data directly comparing early and late CNS metastases are limited. Demleitner et al. examined the molecular profiles of lung carcinoma patients that initially presented with CNS metastases, corresponding to early metastasis in our cohort. In that study, the researchers noted 4% *EGFR* and 57% *KRAS* mutations in the 23 patients evaluated and *ALK* alteration based on FISH in 1.9% (3/157 patients) [22]. The differences between our results may be attributable to variations in population, cohort size, and methodology (RNA NGS vs. in situ hybridization), amongst others.

While our study evaluated DNA and RNA NGS of early versus late CNS metastases, others have performed various analyses to identify differences between NSCLC with and without CNS metastases. For example, variations in the expression of genes such as *CDKN2A*, *ARL9*, *MYC*, *YAP1*, and *MMP13*, amongst others, and in *PI3K* signaling pathways have been noted [1,3,23,24]. Additionally, the presence of less common pathogenic *EGFR* mutations in CNS metastases [3] and immunohistochemical loss of *SMARCA2* have been linked more frequently with CNS metastases [22].

These findings underscore the need for further research to understand the underlying mechanisms and to develop targeted therapeutic strategies.

### 4.4. Limitations

Our study has several limitations. First, the small cohort size may affect the generalizability of our findings. Second, there is a potential selection bias due to its retrospective nature, exclusion of cases with insufficient tissue, and non-adenocarcinoma histology. Third, though patients with early CNS metastases may be considered “treatment naïve”, we did not correlate late CNS metastases with initial stage or management, such as surgery, systemic treatment, or radiation. Fourth, the initial presenting symptoms for some patients were unavailable. Next, the distribution and frequencies of genomic alterations in our cohort may not accurately reflect that across all NSCLC CNS metastases, especially those in the late group, as some patients may not undergo CNS sampling either due to more manageable or significantly advanced disease. Other patient samples may not undergo mutational testing or repeat testing following a previous molecularly characterized primary NSCLC.

### 4.5. Future Directions

Most patients with lung carcinoma present with advanced-stage and generally more aggressive disease at the time of diagnosis [25]. Our results suggest two presentations of CNS metastases: early and late. While no statistically significant differences were noted with DNA and RNA NGS, further prospective and multi-institutional exploration of the two cohorts through larger sample size, more epidemiological studies, and greater genomic profiling, including for the presence of early divergence of clones from the primary carcinoma destined for CNS metastases, could provide valuable insights [26].

## 5. Conclusions

In conclusion, this study highlights the importance of combining DNA NGS with reflex RNA NGS for genomic profiling for CNS metastases in the context of NSCLC. The distinct early and late CNS metastases call for more comprehensive research to unravel the mechanistic differences between these clinical presentations and metastases in general to improve patient outcomes.

## Figures and Tables

**Table 1:** Patients’ Demographics. Targetable DNA Mutations, RNA Alterations, and No Mutations or Alterations.

Variable	Total *n* = 58	Driver Mutation (DNA NGS) *n* = 34 (58.6%)	Driver Alterations (RNA NGS) *n* = 10 (17.2%)	No Driver Mutation or Alteration *n* = 14 (24.1%)	*p*-Value
Age, mean (SD)	66.9 (10.9)	68.7 (8.9)	63.5 (16.3)	64.8 (10.6)	0.30
Male	31 (53.4%)	14 (41.2)	8 (80.0)	9 (64.3)	0.06
Smoking ^1^	40 (72.7%)	23 (69.7)	4 (50.0)	13 (92.9)	0.08
White race ^2^	26 (51.0%)	15 (46.9)	2 (28.6)	9 (75.0)	0.11
Hispanic ^3^	18 (40.9%)	13 (43.3)	2 (33.3)	3 (37.5)	0.88
History of other cancer types—total number ^4^	18 (32.7)	12 (36.4)	1 (12.5)	5 (35.7)	0.42

^1^ Smoking history was unavailable for 3 patients. ^2^ Seven patients did not provide race. ^3^ Fourteen patients did not provide ethnicity. ^4^ Number of patients with ≥1 prior or concurrent non-pulmonary carcinoma(s).

**Table 2:** Mutations: Early versus Late CNS Metastases.

Variable	Total (*n* = 58)	Early CNS Metastasis (*n* = 32)	Late CNS Metastasis (*n* = 26)	*p*-Value
Any Alteration	44 (75.9%)	27 (84.4%)	17 (65.4%)	0.093
DNA	34 (58.6%)	21 (65.6%)	13 (50.0%)	0.36
*EGFR* *L858R* ^1^ *Exon 19 del* *Exon 20 ins*	17 11 5 1	11 8 ^1^ 2 1	6 3 ^2^ 3	
*KRAS* *G12C* *G12D* *G12V* *G12A* *G13R* *Q61H*	15 6 3 3 1 1 1	8 3 2 2 1 1	7 3 1 1 1 - -	
*MET 14 skipping*	2	2		
RNA	10 (17.2%)	6	4	0.74
*ALK*	7	4 ALK::EML4	3 ALK::EML4 (2) ALK::TFG (1)	
*MET 14 skipping*	2	2	0	
*ROS1*	1		1 ROS1::CD74	
No driver alterations	14 (24.1%)	5 (15.6%)	9 (34.6%)	0.093

^1^ *EGFR* T790Mco-occurred with L858R in one early CNS metastasis patient. ^2^ *EGFR* A289T and E709A co-occurred with L858R, each in one patient with late CNS metastasis.

## Data Availability

The original contributions presented in this study are included in the article/Supplementary Material. Further inquiries can be directed to the corresponding author.

## Supplementary Materials

The following supporting information can be downloaded at https://www.mdpi.com/article/10.3390/jpm15050181/s1; Table S1: Patients’ Characteristics: Driver (DNA + RNA) and No Mutations. Table S2: Patients’ Characteristics: Early versus Late CNS Metastases. Table S3: Mutations Early versus Late CNS Metastases, Additional Variants. Figure S1: Cohort of Lung Carcinomas Profiled During Same Timeframe as CNS Metastases.

## Abbreviations

The following abbreviations are used in this manuscript:

NSCLC	Non-small cell lung carcinoma
CNS	Central nervous system
NGS	Next-generation sequencing
IHC	Immunohistochemistry
FISH	Fluorescent in situ hybridization
EGFR	Epidermal growth factor receptor
KRAS	Kirsten rat sarcoma viral oncogene homolog
ALK	Anaplastic lymphoma kinase
MET	MET proto-oncogene, receptor tyrosine kinase
ROS1	c-ros oncogene 1
RET	Rearranged during transfection proto-oncogene
ERBB2	Erb-B2 receptor tyrosine kinase 2 (also known as HER2)
STK11	Serine/threonine kinase 11 (also known as LKB1)
CDKN2A	Cyclin-dependent kinase inhibitor 2A
ARL9	ADP-ribosylation factor-like 9
MYC	MYC proto-oncogene, BHLH transcription factor
YAP1	Yes-associated protein 1
MMP13	Matrix metallopeptidase 13
SMARCA2	SWI/SNF-related, matrix-associated, actin-dependent regulator of chromatin, subfamily A, member 2
PI3K	Phosphoinositide 3-kinase
