# Supplementary material for: Central Nervous System Metastases from Primary Lung Carcinoma: Significance of RNA Fusion Testing and Early Versus Late Metastases
Source: J Pers Med. 2025 May 1;15(5):181. doi: 10.3390/jpm15050181 (PMC12112828; doi:10.3390/jpm15050181)

Supplementary Materials

Supplemental Table S1. Patients' Characteristics. Driver (DNA + RNA) and No Mutations

Variable	Total n = 58	Driver Alterations n = 44	No Driver Alterations n = 14	p-value
Age, mean (SD)	66.9 (10.94)	67.5 (11.08)	64.8 (10.6)	0.21
Male	31 (53.4%)	22 (50.0)	9 (64.3)	0.87
Smoking[1]	40 (72.7%)	27 (65.9)	13 (92.9)	0.05
White race[2]	26 (51.0%)	17 (43.6)	9 (75.0)	0.06
Hispanic/Latino[3]	18 (40.9%)	15 (41.7)	3 (37.5)	0.82
History of other cancer types—total number [4]	18 (32.7%)	13 (31.7)	5 (35.7)	0.78

[1]Smoking history was unavailable for three patients. [2]Seven patients declined history of race. [3]Ethnicity was declined by 14 patients. [4]Number of patients with ≥1 prior or concurrent non-pulmonary carcinoma(s).

Supplemental Table S2. Patients' Characteristics. Early versus Late CNS Metastases.

Variable	Total (n = 58)	Early CNS Metastasis (n = 32)	Late CNS Metastasis (n = 26)	p-value
Age, median (IQR)	66.5 (57.0)	68.5 (35.00)	65.0 (57.0)	0.5
Male (%)	31 (53.4)	17 (53.1)	14 (53.8)	0.95
Smoking (%)[1]	40 (72.7)	21 (72.4)	19 (73.1)	0.95
Race (%)[2]				0.021
• White	26 (51.0)	10 (38.5)	16 (61.5)	
• Black or African American	6 (11.8)	5 (83.3.)	1 (16.7)	
• Asian	6 (11.8)	3 (50.0)	3 (50.0)	
• Other	13 (25.5)	11 (84.6)	2 (15.4)	
Ethnicity (%) Hispanic/Latino [3]	18 (40.9)	11 (47.8)	7 (33.3)	0.32
History of other cancer types (%)[4]	18 (32.7)	8 (26.7)	10 (40.0)	0.3
Reason for lung cancer diagnosis[5]				< 0.001
• Incidental	9 (18.8)	0 (0)	9 (100)	
• Symptomatic	39 (81.3)	30 (76.9)	9 (23.1)	
Any mutations/alterations	44 (75.9)	27 (84.4)	17 (65.4)	0.09

[1]Smoking history was unavailable for three patients. [2]Seven patients declined history of race. [3]Ethnicity was declined by 14 patients. [4]Number of patients with one or more history of

concurrent carcinoma other than lung cancer. [5]Reason for lung cancer diagnosis was unavailable for 10 patients.

Supplemental Table S3. Mutations Early versus Late CNS Metastases, Additional Variants

Mutation	Total (n = 16)	Early CNS Metastasis (n = 8)	Late CNS Metastasis (n = 8)
EGFR			
• A289T			
• T790M		1	2
• E709A	3	-	1
		1	1
(all co-occurred with L858R)			
PIK3CA			
• H1047L			
• E81K: co-occurring		1	1
with EGFR	2	-	1
		1	-
STK11	7	4	3
• P281fs	3	2	1
• D194H	1	-	1
• Q305*	1	1	-
• K78_L80del	1	-	1
• V116fs	1	1	
KEAP1 V561L			
	1	-	1
Co-occurring with EGFR			
POLD1 A354V			
Co-occurring with *ALK* fusion	1	-	1
BRAF N581S	1	1	-

Supplemental Figure S1. Cohort of Lung Carcinomas Profiled During Same Timeframe as CNS Metastases. Total cohort and all alterations.

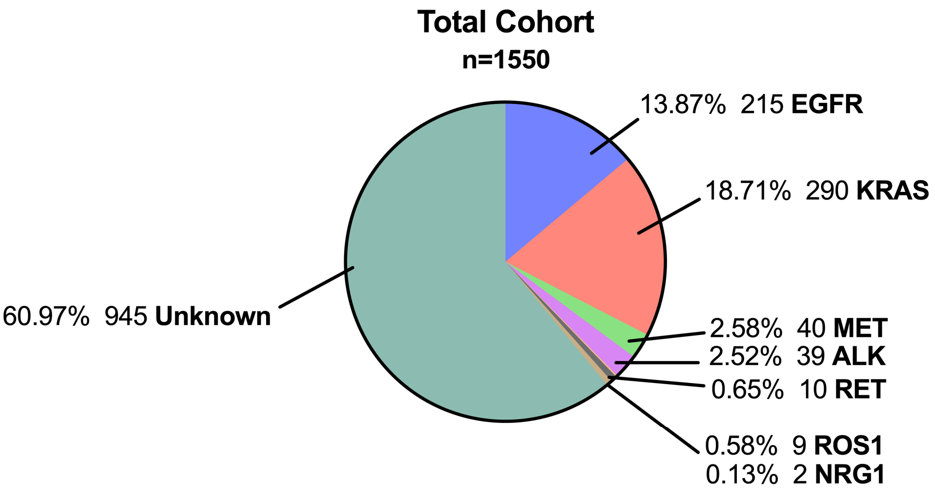

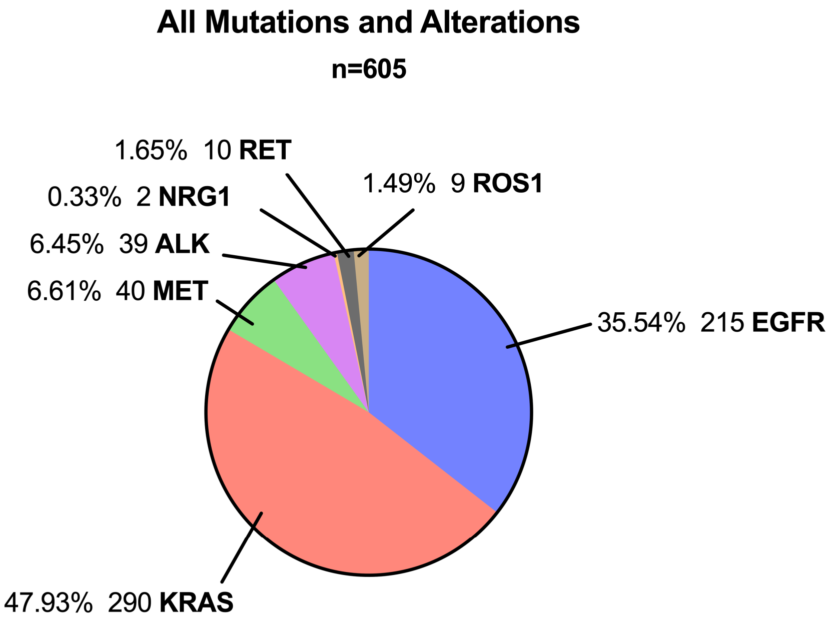

Supplement: Supplementary file 1 [file jpm-15-00181-s001.zip › jpm-3522909-supplementary.pdf]
